# How to perform and evaluate a myocardial perfusion imaging by computed tomography

**DOI:** 10.1093/ehjimp/qyaf001

**Published:** 2025-01-08

**Authors:** Saima Mushtaq, Andrea Baggiano, Francesco Cannata, Alberico Del Torto, Fabio Fazzari, Laura Fusini, Daniele Junod, Riccardo Maragna, Luigi Tassetti, Alessandra Volpe, Nazario Carrabba, Edoardo Conte, Marco Guglielmo, Lucia La Mura, Valeria Pergola, Roberto Pedrinelli, Pasquale Perrone Filardi, Andrea Igoren Guaricci, Gianluca Pontone

**Affiliations:** Perioperative Cardiology and Cardiovascular Imaging Department, Centro Cardiologico Monzino IRCCS, Via C. Parea 4, Milan 20138, Italy; Perioperative Cardiology and Cardiovascular Imaging Department, Centro Cardiologico Monzino IRCCS, Via C. Parea 4, Milan 20138, Italy; Perioperative Cardiology and Cardiovascular Imaging Department, Centro Cardiologico Monzino IRCCS, Via C. Parea 4, Milan 20138, Italy; Perioperative Cardiology and Cardiovascular Imaging Department, Centro Cardiologico Monzino IRCCS, Via C. Parea 4, Milan 20138, Italy; Perioperative Cardiology and Cardiovascular Imaging Department, Centro Cardiologico Monzino IRCCS, Via C. Parea 4, Milan 20138, Italy; Perioperative Cardiology and Cardiovascular Imaging Department, Centro Cardiologico Monzino IRCCS, Via C. Parea 4, Milan 20138, Italy; Perioperative Cardiology and Cardiovascular Imaging Department, Centro Cardiologico Monzino IRCCS, Via C. Parea 4, Milan 20138, Italy; Perioperative Cardiology and Cardiovascular Imaging Department, Centro Cardiologico Monzino IRCCS, Via C. Parea 4, Milan 20138, Italy; Perioperative Cardiology and Cardiovascular Imaging Department, Centro Cardiologico Monzino IRCCS, Via C. Parea 4, Milan 20138, Italy; Perioperative Cardiology and Cardiovascular Imaging Department, Centro Cardiologico Monzino IRCCS, Via C. Parea 4, Milan 20138, Italy; Department of Cardiothoracovascular Medicine, Azienda Ospedaliero-Universitaria Careggi, Florence, Italy; Department of Clinical Cardiology and Cardiovascular Imaging, Galeazzi-Sant'Ambrogio Hospital IRCCS, Milan, Italy; Department of Cardiology, Division of Heart and Lungs, University Medical Center Utrecht, Utrecht University, Utrecht, The Netherlands; Department of Advanced Biomedical Sciences, University Federico II of Naples, Naples, Italy; Department of Cardiac, Thoracic and Vascular Sciences and Public Health, University of Padova, Padova, Italy; Cardiac, Thoracic and Vascular Department, University of Pisa, Pisa, Italy; Department of Advanced Biomedical Sciences, University Federico II of Naples, Naples, Italy; University Cardiology Unit, Interdisciplinary Department of Medicine, University of Bari Aldo Moro, Bari, Italy; Perioperative Cardiology and Cardiovascular Imaging Department, Centro Cardiologico Monzino IRCCS, Via C. Parea 4, Milan 20138, Italy; Department of Biomedical, Surgical and Dental Sciences, University of Milan, Milan, Italy

**Keywords:** coronary artery disease, myocardial perfusion, computed tomography

## Abstract

Stress computed tomography perfusion (CTP) delivers a comprehensive evaluation of both the anatomical and functional aspects in a single examination. It stands out as the only non-invasive technique capable of quantifying coronary stenosis and assessing its functional impact, offering a consolidated diagnostic and management approach for patients with confirmed or suspected coronary artery disease (CAD). This very practical review (‘How to..’ approach) provides guidance on conducting and interpreting static and dynamic CTP, along with an analysis of the strengths and limitations of these methodologies.

## Graphical Abstract

**Graphical abstract:**
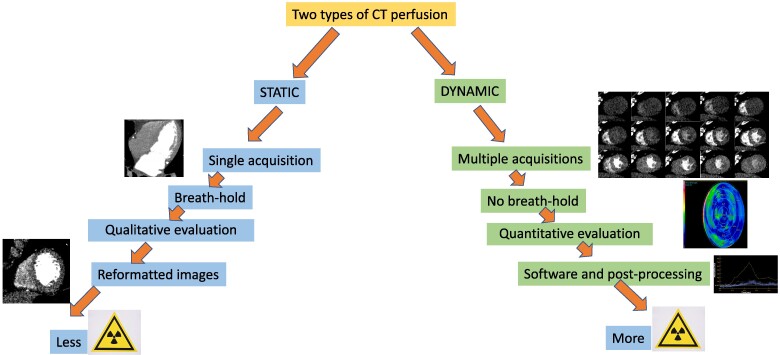

## Introduction

The most appropriate and comprehensive diagnostic flow chart for patients with stable coronary artery disease (CAD) is an evolving and still unresolved matter of debate. Anatomical imaging of the coronary arteries using computed tomography angiography (CCTA) has a very high negative predictive value in patients with low-to-intermediate risk of its diagnostic and prognostic role to rule out CAD with low radiation exposure, and these are the main reasons for its recommendation as an early test to rule out disease.^1–4^ In contrast, cases of patients with known or previously revascularized CAD are significantly more complex, challenging the role of non-invasive coronary artery imaging. In these patients, the addition of functional information is prognostically useful^5^ and in patients with previous history of percutaneous coronary intervention (PCI), functional strategy has been shown to be more cost-effective as compared to anatomical assessment.^6^

CT myocardial perfusion imaging (CTP) has been demonstrated as a technique offering a unique and comprehensive tool for the evaluation of patients with stable chest pain, providing information on coronary atherosclerosis and myocardial perfusion during the same session.^7,8^ CTP has been validated against a clinically well-accepted approach [combined invasive coronary angiography (ICA) and single-photon emission computed tomography (SPECT)],^9^ SPECT,^10^ stress cardiac magnetic resonance,^11^ and invasive fractional flow reserve (FFR).^12^

Imaging of myocardial perfusion with CT was performed as early as the 1980s, but its routine clinical use has remained limited, due to technical issues that could not be addressed with earlier CT technology.^13–15^

The technical improvements of CT scanners, such as increased temporal and spatial resolution and increased coverage, have renewed in the latest years the interest in performing myocardial perfusion CT imaging.^16–18^

There are two main methods for CTP acquisition: static and dynamic myocardial CTP that will be discussed in this practical review (‘How to..’ approach).

## How to prepare patients

**Figure 1:**
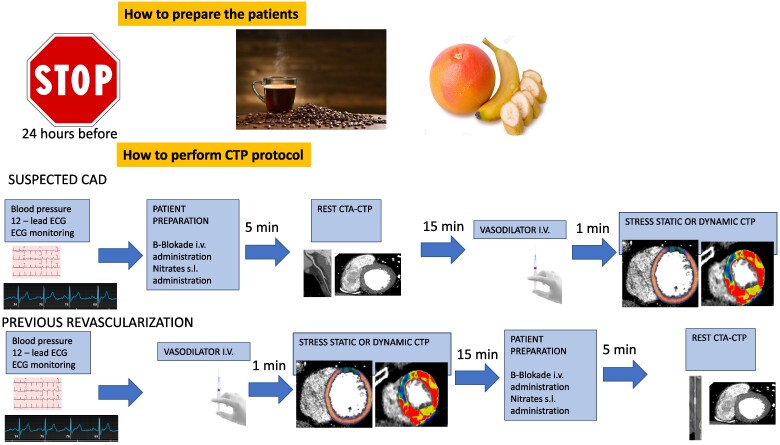
How to prepare patients and how to perform a CT perfusion protocol. *Figure 1* describes in the upper panel what we need to prepare the patients for a CTP study (we need to stop caffeine and some foods such as bananas 24 h before the study) In the lower panel, it has been described the flow in suspected CAD (rest before stress acquisition) and in revascularized patients (stress before the rest).

ECG monitoring and measurements of blood pressure are performed. Two intravenous (IV) lines are essential for CTP studies: one for contrast injection and another for the administration of the vasodilator agent (adenosine or dipiridamole). If regadenoson is used, then we need only one venous access. Patients are asked to observe a fast for 6 h before the scan and to refrain from taking caffeine for 24 h prior to the scan. Caffeine is a competitive inhibitor of the adenosine receptor, and thus, its ingestion can lead to suboptimal pharmacological stress and a false negative scan. Prior to rest CTCA, rate-limiting medication such as beta-blockers may be required to optimize the heart rate and sublingual glyceryl tri-nitrate can be administered to improve visualization of the coronary arteries. *Figure 1* describes how to prepare patients (upper panel). *Table 1* describes the main differences among the vasodilators in terms of mechanism of action, half-life, and way of infusion.

**Table 1:** Stress agents used for the evaluation of myocardial perfusion

Stress agent	Intravenous access	Mechanism of action	Method and timing of infusion	Half-life	Side effects	Cost
Adenosine	Double access	Coronary vasodilatation induced by not-selective A1 adenosine-receptor stimulation (increasing cellular cAMP levels)	Continuous infusion at a dose of 0.14 mg/kg per minute over 3–5 min	10 s	Facial flushing diaphoresis nausea asthma bradyarrhythmias	High
Dypiridamole	Double access	Coronary vasodilatation induced by increasing cellular cAMP levels by inhibition of the phosphodiesterase enzymes that normally break down cAMP	Continuous infusion at a dose of 0.56 mg/kg over 4–6 min	25–30 min (need the antidote aminophylline)	Facial flushing diaphoresis nausea asthma bradyarrhythmias	Low
Regadenoson	Single access	Coronary vasodilatation induced by selective A2A adenosine-receptor stimulation	Intravenously bolus infused in 10 s followed by a saline chaser	2–3 min	Not contraindicated in asthma; headache, dizziness, nausea, stomach discomfort, decreased sense of taste, mild chest discomfort, or warmth, redness, or tingly feeling under your skin	Intermediate–high Not widely available

## Contrast media injection protocol

For both rest and stress CT scans, an intravenous low-iodine contrast agent has to be administered using a power injector. The contrast volume is related to the patient’s weight and the type of scanner used, ranging from 60 to 120 mL in single-energy CT scanners. It can be significantly reduced if dual-energy CT (DECT) technique is applied or if a wide-coverage CT scanner is used (50 mL). The contrast bolus should be delivered with a total injection time of 10 s or less. It is recommended to inject a saline chaser (40–50 mL) following the iodine contrast bolus. CT perfusion techniques are used to image the transit of contrast material from the coronary arteries to the myocardium. Because iodinated contrast material attenuates X-rays proportional to the concentration of iodine, hypoattenuated areas in the myocardium represent myocardial regions of hypoperfusion and/or reduced intravascular blood volume.

## How to perform CT myocardial perfusion

In *Figure 1* (lowers two panels), the workflow of the CTP protocol is described in a subset of patients with suspected CAD and previous revascularization.

Two CT scans have to be carried out: one with pharmacological stress and another in rest condition. A third, optional, delayed CT acquisition can be performed to complete the CT perfusion protocol for the discrimination between viable and nonviable myocardium. The decision to select the opening scan (rest or stress) depends on the pre-test probability of CAD, previous revascularization, and/or the extent of calcium score. It is better to start with the stress phase among patients with intermediate-to-high pre-test and with moderate-to-high calcium scores, and in patients with previous revascularization, as ischaemia needs to be prioritized. When stress CT imaging is performed first, the myocardium is not contaminated by any previous contrast media and so the detection of ischaemia is optimized. On the other hand, it is preferred to initiate the rest phase if the patient has a low to intermediate probability of CAD with no calcium or mild calcium in the coronary arteries, as it is highly probable that the patient has normal coronary arteries, and the high negative predictive value of the CCTA will allow to rule out CAD and thus won’t require to continue with the stress phase.

## Static CTP

### How to acquire static CTP

Static myocardial CTP, which has been more extensively investigated, refers to the assessment of myocardial perfusion obtained from a single data sample of contrast enhancement acquired during the first-pass enhancement of CTCA.^19^ Static CT perfusion imaging is highly dependent on the contrast material bolus timing.

### How to evaluate static CTP

Visual assessment of CT perfusion images is the most common approach for qualitative assessment of myocardial perfusion. The normal myocardium enhances homogeneously after the injection of intravenous contrast material. During the first pass, contrast material diffuses to the interstitial space with a homogeneous incremental increase of myocardial signal intensity over time, which is directly proportional to the iodine content in the tissue. Normal left ventricular myocardial enhancement demonstrates substantially lower attenuation in the lateral wall when compared with the anterior, septal, and inferior walls in patients with normal coronary arteries. The lateral myocardial wall is located adjacent to the air within the lungs and is not subjected to the same beam-hardening effect as the inferior and the septal myocardium, which is located near more dense thoracic structures. This may result in a lower attenuation.^20^ The myocardium will be evaluated on short-axis (apical, medium, and basal slices) and long-axis views (two-, three- and four-chamber projections) with 4- and 8-mm-thick average multiplanar reformatted images. A narrow window width and level (200–350W and 150–200 L) is recommended for perfusion defect evaluation. Each myocardial segment will be correlated to the specific coronary territory as described by Cerci *et al*.^21^ Myocardial analysis consists of the evaluation of the myocardium in short-axis views from the apex to the base of the left ventricle, using the 17 segments suggested by the American Heart Association classification.

Static CTP can be semiquantitatively analysed using myocardial CT attenuation in each myocardial segment or subendocardial or epicardial layer.^22^ The transmural perfusion ratio (TPR), defined as the ratio of segment-specific subendocardial attenuation to subepicardial attenuation, has been introduced as a quantitative index of static CTP.^23^ However, more recent studies demonstrated that visual assessment of static CTP provides superior diagnostic performance over the TPR.^22,24^

### Strengths and limits of static CTP

Static CT perfusion imaging that is based on a single acquisition after injection of the stress agent is highly dependent on the contrast material bolus timing. A drawback of static CT perfusion is that the peak attenuation may be missed because only one sample of data is acquired.

Another challenge of myocardial CTP is represented by beam-hardening artefacts which could be misinterpreted as perfusion defects.^25^ With the development of dual-energy CT, beam-hardening artefacts can be attenuated or even cancelled by the generation of monochromatic images at medium-to-high energy levels. Static CT perfusion imaging has been performed by using the dual-energy mode.^26^ In a static CTP protocol, a review of multiple cardiac phase images can help to distinguish true perfusion defects from motion or beam-hardening artefacts.^11,22^ True perfusion defects are distributed among the coronary territories, from the subendocardial to the transmural myocardial wall.^27^ In addition, true perfusion defects may persist on stress images throughout all cardiac phases, from systolic to diastolic. Unlike true perfusion defects, motion or beam-hardening artefacts do not correspond to a coronary territory and might appear in only one or two cardiac phases.^27^ A common location of beam-hardening artefacts is the basal inferior wall, likely due to the nearby enhancing descending aorta and vertebral bodies.

Static CTP may underestimate myocardial hypoperfusion in case of balanced ischaemia (i.e. multivessel coronary artery disease) as it enables only qualitative, or at most semi-quantitative evaluation of myocardial perfusion^28^

**Figure 2:**
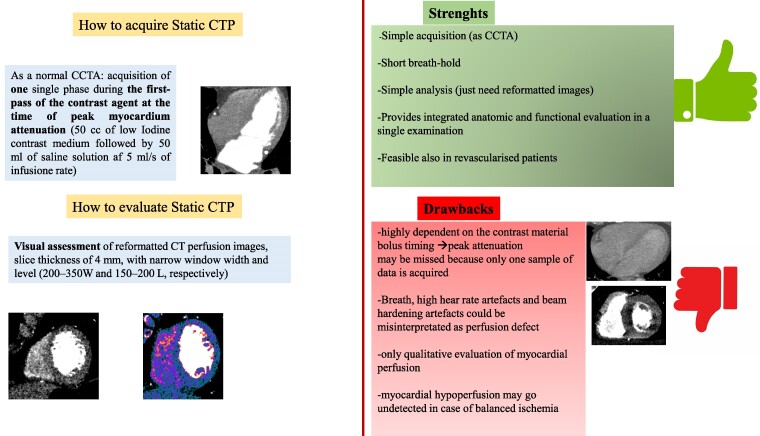
How to: static CTP. *Figure 2* describes in the left panel how to acquire and evaluate a static CTP and in the right panel the main strengths and drawbacks of this method.

*Figure 2* shows how to acquire and evaluate static CTP and the main strengths and limits of this perfusion approach.

### Analysis of the current literature for static CTP

The diagnostic accuracy of CTP has been compared with that of other non-invasive imaging modalities, including SPECT, MRI, and positron emission tomography (PET).^10,29,30^ CTP provides incremental benefit for the diagnosis of haemodynamically significant stenosis defined by FFR, particularly by improving specificity.^22,30^ In a meta-analysis, CTP showed comparable diagnostic accuracy to MRI and PET and was better than SPECT for the detection of myocardial ischaemia defined by FFR.^8^ In a meta-analysis performed by Pelgrim *et al*., CTP showed acceptable diagnostic performance, with a sensitivity ranging from 75% to 89% and specificity from 78% to 95% compared with invasive angiography, SPECT, or MRI.^31^

The CORE320 study, which is the largest study of CTP thus far performed, compared the diagnostic performance of static CTP acquired by a wide-detector scanner in 381 patients with that of combined SPECT and invasive coronary angiography.^9^ In that study, the combination of CCTA and CTP correctly identified patients with flow-limiting coronary stenosis. In a substudy of CORE320, CTP showed higher diagnostic accuracy than SPECT in predicting obstructive CAD on quantitative invasive coronary angiography.^10^ Recently, the PERFECTION study evaluated the diagnostic accuracy of CTP, performed with a whole-heart coverage CT scanner for the detection of functionally significant CAD by using ICA plus invasive FFR as the reference standard in 100 consecutive intermediate- to high-risk symptomatic patients authors concluded that the inclusion of stress CTP for the evaluation of patients with an intermediate to high risk of CAD improved the diagnostic performance of CCTA for detecting functionally significant CAD.^12^ CCTA alone demonstrated a per-vessel and per-patient specificity, positive predictive value, and accuracy of 76%, 63%, and 98%, and 54%, 68%, and 76%, respectively. Combining CCTA with static stress CTP, per-vessel, and per-patient specificity, positive predictive value, and accuracy significantly improve in both models (94%, 86%, 93% and 83%, 86%, 91%).

The CATH2 was a randomized controlled trial aimed to evaluate the clinical efficacy of combined examination with CCTA and CTP compared to CCTA alone in 300 patients hospitalized for acute-onset chest pain.^32^ Patients were randomized 1:1 to examination with coronary CTA or coronary CTA + CTP. The primary endpoint was the frequency of coronary revascularization among patients referred for ICA based on index computed tomography evaluation. A post-discharge diagnostic strategy of coronary CTA + CTP safely reduces the need for invasive examination and treatment in patients suspected of having ischaemic heart disease. Static CTP may also improve CCTA diagnostic accuracy in patients who underwent prior percutaneous intervention with coronary stenting. Rief *et al*. showed that a combined CCTA + CTP protocol improved cardiac CT diagnostic rate and accuracy compared with CCTA alone (100% vs. 78% and 87% vs. 71% at the patient level and vessel level, respectively) in 20 patients previously treated with coronary stenting.^33^ Similarly, Magalhaes *et al*. reported a diagnostic accuracy in the territory-based analysis of 91% for CCTA + CTP evaluation vs. 77% for CCTA alone in 46 patients.^34^ The ADVANTAGE prospective study evaluated 150 patients with previous coronary stent implantation and demonstrated a diagnostic accuracy of CTP significantly higher than that of CCTA in the territory-based and patient-based analysis (92.1% vs. 85.6% and 86.7% vs. 76.7%, respectively) when quantitative coronary angiography was used as a gold standard. Similarly, CTP specificity and diagnostic accuracy were significantly higher than those of CCTA when invasive FFR was employed as a gold standard. Of note, the radiation exposure of cardiac CT (CCTA + CTP) was 4.15 ± 1.5 mSv.^35^

## Dynamic CTP

### How to acquire dynamic CTP

Patients preparation, pharmacological stress protocol, and contrast administration do not differ from static CT perfusion; on the contrary, while for static CTP, only one scan is performed at the time of peak myocardium contrast attenuation, for dynamic CTP, during intravenous contrast medium injection, repeated rapid CT scans are acquired to allow determination of time–attenuation curves (TACs). Indeed, dynamic CTP refers to the assessment of myocardial perfusion based on multiple samples of myocardial attenuation at sequential time points of contrast enhancement after injection of a short contrast material bolus to create time–attenuation curves allowing us to quantify myocardial blood flow (MBF). More specifically approximately 20–25 CT scans are performed during contrast medium wash-in and wash-out through the myocardium while patients breath normally; thus, respiratory motion correction is needed in order to reduce motion artefacts. From TACs, a value of myocardial flow is then obtained through different methods; all techniques are based on the dynamic change of attenuation values recorded on the specific regions of interest, which are proportional to the concentration of contrast material in the myocardium and of consequence to myocardial blood flow, even if the relationship between contrast medium retention and myocardial perfusion is not linear and mathematical modelling are needed.^36–38^

### How to evaluate dynamic CTP

After injection of a stress agent repeated rapid CT scans need to be acquired during intravenous contrast medium injection to allow determination of time-attenuation curves (TACs). From TACs, a value of MBF is then obtained through different methods, all based on the dynamic change of attenuation values, that are proportional to the concentration of contrast material in the myocardium and of consequence to MBF.^36–38^ In particular, orientation of three- and two-chamber views and short-axis view are obtained. A region of interest in the ascending aorta is placed for arterial impact function (AIF) calculation. References of basal anterior and inferior wall and apex are placed for myocardial segmentation in a standard 17-segment model, with endocardial and epicardial borders. For quantification of MBF, myocardial time/attenuation curves are coupled with the AIF using a hybrid deconvolution model. MBF maps were reconstructed as a stack of colour-coded images with a slice thickness of 3.0 mm. Measurements of MBF are automatically calculated as a mean value usually expressed as mL/100 mL/min in every single myocardial segment; point-by-point value is also available. The value of hyperaemic MBF of 101 mL/100 g/min was used as an optimal threshold for absolute MBF to identify functionally significant CAD, as previously validated.^39^ Nevertheless, substantial divergences in the reported optimal cut-off values and diagnostic accuracy for MBF calculated from CTP datasets have been reported.^40–43^ This may be caused not only by technical or methodologic reasons but also by systematic interindividual differences in MBF. As recently reported, a relative measure of focal MBF in comparison with healthy remote myocardium (MBF ratio) may be more accurate for the identification of haemodynamically significant coronary artery stenosis.^44^ A multicentre study indicated that the assessment of MBF ratio allows superior discrimination of significant coronary artery stenosis compared with absolute MBF in patients undergoing coronary CTA and CTP.^45^

**Figure 3:**
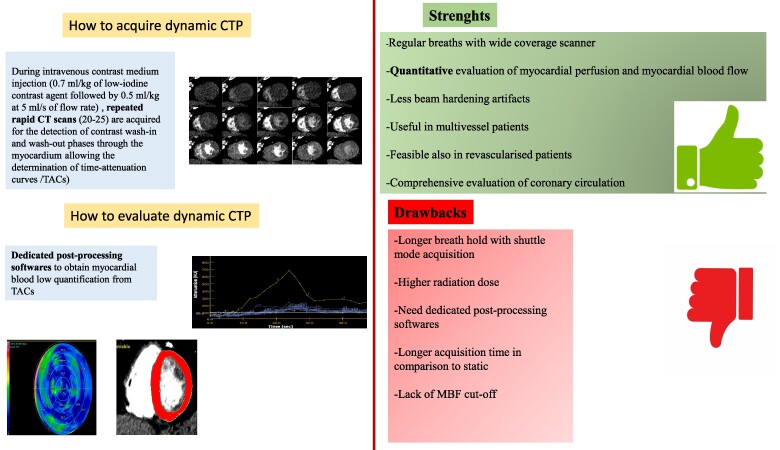
How to: dynamic CTP. *Figure 3* describes in the left panel how to acquire and evaluate a dynamic CTP and in the right panel the main strengths and drawbacks of this method.

Analysis of the MBF ratio may help overcome diagnostic limitations due to methodologic differences or interindividual MBF variability and may allow a more robust identification of patients with myocardial ischaemia. *Figure 3* showed how to acquire and evaluate dynamic CTP and the main strenght and limits of this perfusion approach.

### Strengths and limits of dynamic CTP

One of the major strengths of dynamic CTP is that is not a subjective and qualitative evaluation of myocardial perfusion but is a quantitative evaluation of perfusion with the calculation of MBF. It does not require breath-hold but regular breaths with a wide-coverage scanner. In comparison with static CTP less beam-hardening artefacts are described although some artefacts (i.e. the presence of very dense structures may cause beam hardening) may result in hypodense areas in myocardium, most commonly on the posterolateral wall, mimicking perfusion defects.^38^

The dynamic approach has a significant advantage over the static modality, which is represented by the fact that it provides a quantitative measurement of the regional MBF compared to a qualitative assessment of myocardial perfusion only. This is particularly useful in the setting of diffuse CAD, where qualitative perfusion assessment is not sensitive enough and may not be sufficient to identify a functionally significant stenosis in the presence of diffuse and balanced ischaemia.^46^ Despite growing evidence for the diagnostic accuracy of CTP, a robust cut-off value for hyperaemic MBF that allows the identification of hemodynamically relevant stenosis is lacking.

One of the main drawbacks of dynamic CTP is the high radiation dose, which is directly correlated with acquisition protocol; more specifically radiation doses of at least 11 mSv and up to 18 mSv have been previously reported, with further increase if coronary anatomy needs to be evaluated as dynamic CTP data set cannot be used for coronary anatomy evaluation and a separate CT scan has to be acquired.^12,43,47,48^ With wide-coverage scanners, the radiation dose was lower (5.63 ± 4.2 mSv for the whole cardiac CT exam, CCTA and CTP).^39^

### Analysis of the current literature of dynamic CTP

Dynamic CTP for MBF quantification was first described in an animal model in 1987.^15^ In 2008, the first human study with 16-slice MDCT compared to myocardial scintigraphy showed promising results; however, it must be underlined that using 16-slice MDCT authors were not able to cover and obtain data from the whole heart.^49^ Similar results were reported in 2012 by So *et al*. with 64-slice MDCT at the expense of high radiation dose (19.4 mSv).^48^ In 2013, Rossi *et al*. suggested that dynamic CTP had high diagnostic accuracy than anatomical evaluation of coronary artery by CCTA when compared with invasive FFR (cut-off of <0.75) using a second-generation CT scanner (AUC, 0.95 vs. 0.89, respectively)^43^

In 2018, a meta-analysis was performed including 13 studies and 482 patients. Most of the studies used adenosine as a stressor agent, and dual-source CT was the most represented scanner type (69%). Authors reported good diagnostic accuracy of dynamic CTP when compared to different reference standard, including invasive FFR for myocardial ischaemia detection; more specifically, sensitivity and specificity of 0.83 and 0.90 at the segment level, and of 0.93 and 0.82 at the patient level, respectively, were reported. However, radiation dose ranged from 5.3 to 10.5 mSv per dynamic perfusion and 9.3 to 18.1 for the entire CT scan protocol, including coronary anatomy evaluation.^47^

To the best of our knowledge, only a few studies addressed the prognostic role of dynamic CTP. In 2017, Meinel FG *et al*. enrolled 144 patients who underwent both CCTA and dynamic CTP; the authors reported that myocardial perfusion CT has incremental predictive value over clinical risk factors and detection of coronary artery stenosis with CCTA.^50^ More recently, CCTA, FFRct, and dynamic CTP were evaluated in a multicentre trial that included 84 patients; authors demonstrated that myocardial blood flow evaluated by dynamic CTP acquisitions has the highest prognostic value, over CCTA and CT-FFR values, in terms of future major adverse cardiac events (cardiac death, nonfatal myocardial infarction, unstable angina requiring hospitalization, or revascularization) at a mean follow-up of 18 months.^51^

Dynamic CTP with last-generation CT scanner may be used for accurate quantification of myocardium blood flow (MBF) and results obtained in recent studies demonstrated that dynamic CTP may have a prognostic role over anatomical evaluation and FFRct.^39^ Also, another multicentre trial (SPECIFIC), using third-generation dual-source CT, investigated the diagnostic performance of dynamic stress CTP in addition to CCTA compared to ICA and FFR and demonstrated that Dynamic CTP offers incremental diagnostic value over CCTA alone for the identification of hemodynamically significant CAD.^52^

Dynamic CTP offers an approach to evaluate the entire cascade of the myocardial perfusion, which translates into a true functional test. This could be particularly important in women and diabetic patients in whom microcirculatory disease could contribute to chest pain. The mean radiation dose ranged from 5.3 for CCTA/static CTP approach to 10.5 mSv for the dynamic perfusion^35,39^.

**Figure 4:**
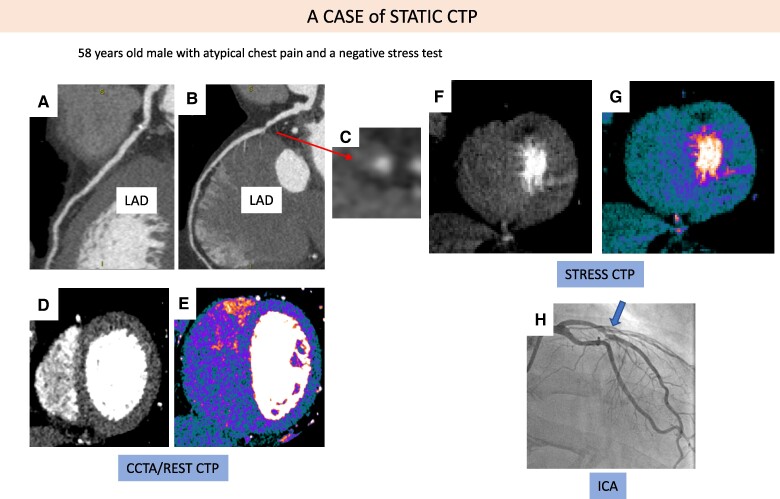
A case of static CTP. A case of 58-year-old male with atypical chest pain and a negative stress test: A 60–70% stenosis of the left anterior descending artery (LAD) was found at CCTA (panels *A–B*). Note the mixed plaque with positive remodelling and low attenuation in the short-axis view (panel *C*). Rest CTP was normal (panels *D–E*), while static stress CTP showed a perfusion defect in the mid-apical portion of the anterior wall (panels *F–G*). At ICA, LAD stenosis was confirmed (panel *H*).

**Figure 5:**
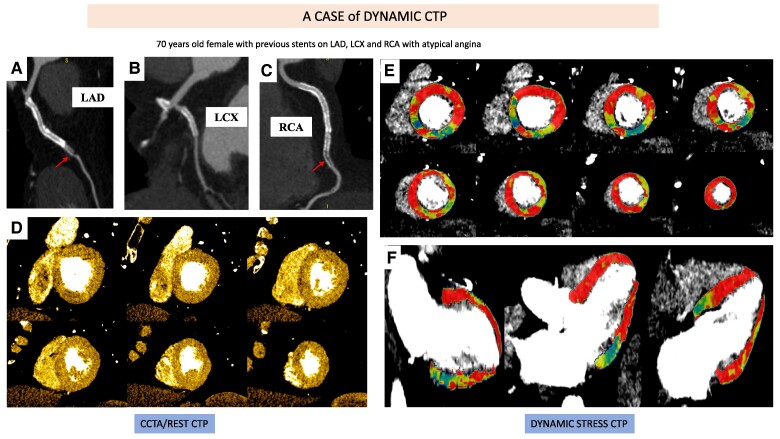
A case of dynamic CTP: 70-year-old female with known CAD (stents on LAD, LCX, and RCA) with atypical chest pain: CTCA showed a restenosis of the stent on mid-distal LAD (arrow, panel *A*), patent stent on LCX (panel *B*) and a restenosis of the stent on RCA (arrow, panel *C*). Rest CTP was normal (panels *D*) from basal until apical portion, while dynamic stress CTP showed a reduction of MBF values in anterolateral and inferolateral wall (panel *E*).

*Figures 4* and *5* show two cases of static CTP and dynamic CTP, respectively.

### Challenging patients (high calcium burden, atrial fibrillation, pacemaker electrodes, and multiple premature beats)

The recent improvements in CT technology with whole coverage detector and increased rotation speed allows to scan also patients in atrial fibrillation and with premature beats in one cardiac beat.^17^ Patients with high calcium burden are considered as high pre-test probability of CAD and usually the stress phase is performed before the rest using a Kvp of 100 and 120. Patients with pacemaker are studied as other patients with same anatomical and clinical characteristics but the presence of electrodes can generate some artefacts especially in the septum and inferior wall of left ventricle.

Low kilovolt scanning, new CT detector technology, as photon counting CT and algorithmic solutions are promising approaches to reducing CT radiation dose and image artefacts, also in paediatric patients^53,54^

## Conclusions

Multicenter and multivendor cost-effectiveness studies are required in order to fix clinical indication for obtaining dynamic myocardial CTP imaging and CTP-PRO trial is the first ongoing randomized trial testing the cost-effectiveness of CCTA + CTP with a detailed analysis of the costs of perfusion imaging^55^ CTP would likely find an ideal role in patients with moderate, diffuse multivessel coronary lesions or patients already revascularized, helping physicians to better triage patients, and reducing the possible over-referral to myocardial revascularization after CCTA.^48,49^

Current evidence suggests that adding static or dynamic CTP imaging is a safe and powerful tool to improve the accuracy and the positive predictive value of CCTA alone because it not only provides anatomic information concerning luminal stenosis, plaque morphology, and total plaque burden but also provides data on myocardial tissue haemodynamics. The combined use of CTA + CTP should be the routine approach for patients with intermediate stenosis in the setting of suspected CAD and in stented patients.

## Data Availability

No new data were generated or analysed in support of this research.

## Lead author biography

Saima Mushtaq is a physician, with a postgraduate in Cardiology in the University of Milan who has dedicated her clinical and scientific activity to Cardiovascular Imaging. Nowadays, she is the Director of Cardiovascular CT under the Post-operative and Cardiovascular Imaging Department of Prof Gianluca Pontone, at Centro Cardiologico Monzino, IRCCS, Milan; she has been councillor of EACVI and vice-chairman of the Italian Group of Cardiac CT. She has more than 200 articles published in peer-reviewed International Journals with Impact Factor regarding Cardiac CT and MRI. She had been part of several multicentre trials. She has the European Certification of MRI (level III) and SCCT Certification for Cardiac CT (Level III).

## References

[1] Erthal F, Premaratne M, Yam Y, Chen L, Lamba J, Keenan M et al Appropriate use criteria for cardiac computed tomography: does computed tomography have incremental value in all appropriate use criteria categories? J Thorac Imaging 2018;33:132–7. 28914747 10.1097/RTI.0000000000000297

[2] Pontone G, Bertella E, Mushtaq S, Loguercio M, Cortinovis S, Baggiano A et al Coronary artery disease: diagnostic accuracy of CT coronary angiography–a comparison of high and standard spatial resolution scanning. Radiology 2014;271:688–94. 24520943 10.1148/radiol.13130909

[3] Pontone G, Muscogiuri G, Baggiano A, Andreini D, Guaricci AI, Guglielmo M et al Image quality, overall evaluability, and effective radiation dose of coronary computed tomography angiography with prospective electrocardiographic triggering plus intracycle motion correction algorithm in patients with a heart rate over 65 beats per Minute. J Thorac Imaging 2018;33:225–31. 29346192 10.1097/RTI.0000000000000320

[4] Task Force M, Montalescot G, Sechtem U, Achenbach S, Andreotti F, Arden C et al 2013 ESC guidelines on the management of stable coronary artery disease: the task force on the management of stable coronary artery disease of the European Society of Cardiology. Eur Heart J 2013;34:2949–3003. 23996286 10.1093/eurheartj/eht296

[5] Pontone G, Andreini D, Bartorelli AL, Bertella E, Cortinovis S, Mushtaq S et al A long-term prognostic value of CT angiography and exercise ECG in patients with suspected CAD. JACC Cardiovasc Imaging 2013;6:641–50. 23764093 10.1016/j.jcmg.2013.01.015

[6] Pontone G, Andreini D, Guaricci AI, Rota C, Guglielmo M, Mushtaq S et al The STRATEGY study (stress cardiac magnetic resonance versus computed tomography coronary angiography for the management of symptomatic revascularized patients): resources and outcomes impact. Circ Cardiovasc Imaging 2016;9:e005171. 27894070 10.1161/CIRCIMAGING.116.005171

[7] Schuijf JD, Wijns W, Jukema JW, Atsma DE, de Roos A, Lamb HJ et al Relationship between noninvasive coronary angiography with multi-slice computed tomography and myocardial perfusion imaging. J Am Coll Cardiol 2006;48:2508–14. 17174190 10.1016/j.jacc.2006.05.080

[8] Takx RA, Blomberg BA, El Aidi H, Habets J, de Jong PA, Nagel E et al Diagnostic accuracy of stress myocardial perfusion imaging compared to invasive coronary angiography with fractional flow reserve meta-analysis. Circ Cardiovasc Imaging 2015;8:e002666. 25596143 10.1161/CIRCIMAGING.114.002666

[9] Rochitte CE, George RT, Chen MY, Arbab-Zadeh A, Dewey M, Miller JM et al Computed tomography angiography and perfusion to assess coronary artery stenosis causing perfusion defects by single photon emission computed tomography: the CORE320 study. Eur Heart J 2014;35:1120–30. 24255127 10.1093/eurheartj/eht488 PMC6693293

[10] George RT, Arbab-Zadeh A, Miller JM, Vavere AL, Bengel FM, Lardo AC et al Computed tomography myocardial perfusion imaging with 320-row detector computed tomography accurately detects myocardial ischemia in patients with obstructive coronary artery disease. Circ Cardiovasc Imaging 2012;5:333–40. 22447807 10.1161/CIRCIMAGING.111.969303

[11] Bettencourt N, Chiribiri A, Schuster A, Ferreira N, Sampaio F, Pires-Morais G et al Direct comparison of cardiac magnetic resonance and multidetector computed tomography stress-rest perfusion imaging for detection of coronary artery disease. J Am Coll Cardiol 2013;61:1099–107. 23375929 10.1016/j.jacc.2012.12.020

[12] Pontone G, Andreini D, Guaricci AI, Baggiano A, Fazzari F, Guglielmo M et al Incremental diagnostic value of stress computed tomography myocardial perfusion with whole-heart coverage CT scanner in intermediate- to high-risk symptomatic patients suspected of coronary artery disease. JACC Cardiovasc Imaging 2019;12:338–49. 29454774 10.1016/j.jcmg.2017.10.025

[13] Bell MR, Lerman LO, Rumberger JA. Validation of minimally invasive measurement of myocardial perfusion using electron beam computed tomography and application in human volunteers. Heart 1999;81:628–35. 10336923 10.1136/hrt.81.6.628 PMC1729070

[14] Rumberger JA, Bell MR. Measurement of myocardial perfusion and cardiac output using intravenous injection methods by ultrafast (cine) computed tomography. Invest Radiol 1992;27:S40–6. 1468874 10.1097/00004424-199212002-00008

[15] Rumberger JA, Feiring AJ, Lipton MJ, Higgins CB, Ell SR, Marcus ML. Use of ultrafast computed tomography to quantitate regional myocardial perfusion: a preliminary report. J Am Coll Cardiol 1987;9:59–69. 3540073 10.1016/s0735-1097(87)80083-9

[16] Andreini D, Mushtaq S, Pontone G, Conte E, Guglielmo M, Annoni A et al Diagnostic performance of coronary CT angiography carried out with a novel whole-heart coverage high-definition CT scanner in patients with high heart rate. Int J Cardiol 2018;257:325–31. 29506722 10.1016/j.ijcard.2017.10.084

[17] Andreini D, Pontone G, Mushtaq S, Conte E, Perchinunno M, Guglielmo M et al Atrial fibrillation: diagnostic accuracy of coronary CT angiography performed with a whole-heart 230-microm spatial resolution CT scanner. Radiology 2017;284:676–84. 28445682 10.1148/radiol.2017161779

[18] Andreini D, Pontone G, Mushtaq S, Mancini ME, Conte E, Guglielmo M et al Image quality and radiation dose of coronary CT angiography performed with whole-heart coverage CT scanner with intra-cycle motion correction algorithm in patients with atrial fibrillation. Eur Radiol 2018;28:1383–92. 29164383 10.1007/s00330-017-5131-2

[19] Mushtaq S, Conte E, Pontone G, Baggiano A, Annoni A, Formenti A et al State-of-the-art-myocardial perfusion stress testing: static CT perfusion. J Cardiovasc Comput Tomogr 2020;14:294–302. 31530496 10.1016/j.jcct.2019.09.002

[20] Crossett MP, Schneider-Kolsky M, Troupis J. Normal perfusion of the left ventricular myocardium using 320 MDCT. J Cardiovasc Comput Tomogr 2011;5:406–11. 22146499 10.1016/j.jcct.2011.10.003

[21] Cerci RJ, Arbab-Zadeh A, George RT, Miller JM, Vavere AL, Mehra V et al Aligning coronary anatomy and myocardial perfusion territories: an algorithm for the CORE320 multicenter study. Circ Cardiovasc Imaging 2012;5:587–95. 22887690 10.1161/CIRCIMAGING.111.970608

[22] Yang DH, Kim YH, Roh JH, Kang JW, Han D, Jung J et al Stress myocardial perfusion CT in patients suspected of having coronary artery disease: visual and quantitative analysis-validation by using fractional flow reserve. Radiology 2015;276:715–23. 25880262 10.1148/radiol.2015141126

[23] George RT, Arbab-Zadeh A, Miller JM, Kitagawa K, Chang HJ, Bluemke DA et al Adenosine stress 64- and 256-row detector computed tomography angiography and perfusion imaging: a pilot study evaluating the transmural extent of perfusion abnormalities to predict atherosclerosis causing myocardial ischemia. Circ Cardiovasc Imaging 2009;2:174–82. 19808590 10.1161/CIRCIMAGING.108.813766 PMC3035629

[24] Ko BS, Cameron JD, Leung M, Meredith IT, Leong DP, Antonis PR et al Combined CT coronary angiography and stress myocardial perfusion imaging for hemodynamically significant stenoses in patients with suspected coronary artery disease: a comparison with fractional flow reserve. JACC Cardiovasc Imaging 2012;5:1097–111. 23153909 10.1016/j.jcmg.2012.09.004

[25] Rodriguez-Granillo GA, Carrascosa P, Cipriano S, De Zan M, Deviggiano A, Capunay C et al Beam hardening artifact reduction using dual energy computed tomography: implications for myocardial perfusion studies. Cardiovasc Diagn Ther 2015;5:79–85. 25774354 10.3978/j.issn.2223-3652.2015.01.13 PMC4329162

[26] So A, Hsieh J, Narayanan S, Thibault JB, Imai Y, Dutta S et al Dual-energy CT and its potential use for quantitative myocardial CT perfusion. J Cardiovasc Comput Tomogr 2012;6:308–17. 23040537 10.1016/j.jcct.2012.07.002

[27] Koo HJ, Yang DH, Kim YH, Kang JW, Kang SJ, Kweon J et al CT-based myocardial ischemia evaluation: quantitative angiography, transluminal attenuation gradient, myocardial perfusion, and CT-derived fractional flow reserve. Int J Cardiovasc Imaging 2016;32:1–19. 10.1007/s10554-015-0825-5 26667445

[28] Danad I, Szymonifka J, Schulman-Marcus J, Min JK. Static and dynamic assessment of myocardial perfusion by computed tomography. Eur Heart J Cardiovasc Imaging 2016;17:836–44. 27013250 10.1093/ehjci/jew044 PMC4955293

[29] Kikuchi Y, Oyama-Manabe N, Naya M, Manabe O, Tomiyama Y, Sasaki T et al Quantification of myocardial blood flow using dynamic 320-row multi-detector CT as compared with (1)(5)O-H(2)O PET. Eur Radiol 2014;24:1547–56. 24744200 10.1007/s00330-014-3164-3

[30] Ko SM, Choi JW, Song MG, Shin JK, Chee HK, Chung HW et al Myocardial perfusion imaging using adenosine-induced stress dual-energy computed tomography of the heart: comparison with cardiac magnetic resonance imaging and conventional coronary angiography. Eur Radiol 2011;21:26–35. 20658242 10.1007/s00330-010-1897-1

[31] Pelgrim GJ, Dorrius M, Xie X, den Dekker MA, Schoepf UJ, Henzler T et al The dream of a one-stop-shop: meta-analysis on myocardial perfusion CT. Eur J Radiol 2015;84:2411–20. 25636388 10.1016/j.ejrad.2014.12.032

[32] Sorgaard MH, Linde JJ, Kuhl JT, Kelbaek H, Hove JD, Fornitz GG et al Value of myocardial perfusion assessment with coronary computed tomography angiography in patients with recent acute-onset chest pain. JACC Cardiovasc Imaging 2018;11:1611–21. 29248654 10.1016/j.jcmg.2017.09.022

[33] Rief M, Zimmermann E, Stenzel F, Martus P, Stangl K, Greupner J et al Computed tomography angiography and myocardial computed tomography perfusion in patients with coronary stents: prospective intraindividual comparison with conventional coronary angiography. J Am Coll Cardiol 2013;62:1476–85. 23792193 10.1016/j.jacc.2013.03.088

[34] Magalhaes TA, Cury RC, Pereira AC, Moreira Vde M, Lemos PA, Kalil-Filho R et al Additional value of dipyridamole stress myocardial perfusion by 64-row computed tomography in patients with coronary stents. J Cardiovasc Comput Tomogr 2011;5:449–58. 22146504 10.1016/j.jcct.2011.10.013

[35] Andreini D, Mushtaq S, Pontone G, Conte E, Collet C, Sonck J et al CT perfusion versus coronary CT angiography in patients with suspected in-stent restenosis or CAD progression. JACC Cardiovasc Imaging 2020;13:732–42. 31422127 10.1016/j.jcmg.2019.05.031

[36] Bamberg F, Marcus RP, Becker A, Hildebrandt K, Bauner K, Schwarz F et al Dynamic myocardial CT perfusion imaging for evaluation of myocardial ischemia as determined by MR imaging. JACC Cardiovasc Imaging 2014;7:267–77. 24529887 10.1016/j.jcmg.2013.06.008

[37] Miles KA, Griffiths MR. Perfusion CT: a worthwhile enhancement? Br J Radiol 2003;76:220–31. 12711641 10.1259/bjr/13564625

[38] Rossi A, Merkus D, Klotz E, Mollet N, de Feyter PJ, Krestin GP. Stress myocardial perfusion: imaging with multidetector CT. Radiology 2014;270:25–46. 24354374 10.1148/radiol.13112739

[39] Pontone G, Baggiano A, Andreini D, Guaricci AI, Guglielmo M, Muscogiuri G et al Dynamic stress computed tomography perfusion with a whole-heart coverage scanner in addition to coronary computed tomography angiography and fractional flow reserve computed tomography derived. JACC Cardiovasc Imaging 2019;12:2460–71. 31005531 10.1016/j.jcmg.2019.02.015

[40] Bucher AM, De Cecco CN, Schoepf UJ, Wang R, Meinel FG, Binukrishnan SR et al Cardiac CT for myocardial ischaemia detection and characterization–comparative analysis. Br J Radiol 2014;87:20140159. 25135617 10.1259/bjr.20140159 PMC4207157

[41] Bamberg F, Becker A, Schwarz F, Marcus RP, Greif M, von Ziegler F et al Detection of hemodynamically significant coronary artery stenosis: incremental diagnostic value of dynamic CT-based myocardial perfusion imaging. Radiology 2011;260:689–98. 21846761 10.1148/radiol.11110638

[42] Ko BS, Cameron JD, Meredith IT, Leung M, Antonis PR, Nasis A et al Computed tomography stress myocardial perfusion imaging in patients considered for revascularization: a comparison with fractional flow reserve. Eur Heart J 2012;33:67–77. 21810860 10.1093/eurheartj/ehr268

[43] Rossi A, Dharampal A, Wragg A, Davies LC, van Geuns RJ, Anagnostopoulos C et al Diagnostic performance of hyperaemic myocardial blood flow index obtained by dynamic computed tomography: does it predict functionally significant coronary lesions? Eur Heart J Cardiovasc Imaging 2014;15:85–94. 23935153 10.1093/ehjci/jet133

[44] Kono AK, Coenen A, Lubbers M, Kurata A, Rossi A, Dharampal A et al Relative myocardial blood flow by dynamic computed tomographic perfusion imaging predicts hemodynamic significance of coronary stenosis better than absolute blood flow. Invest Radiol 2014;49:801–7. 25014013 10.1097/RLI.0000000000000087

[45] Wichmann JL, Meinel FG, Schoepf UJ, Lo GG, Choe YH, Wang Y et al Absolute versus relative myocardial blood flow by dynamic CT myocardial perfusion imaging in patients with anatomic coronary artery disease. AJR Am J Roentgenol 2015;205:W67–72. 26102420 10.2214/AJR.14.14087

[46] Nissen L, Winther S, Westra J, Ejlersen JA, Isaksen C, Rossi A et al Diagnosing coronary artery disease after a positive coronary computed tomography angiography: the Dan-NICAD open label, parallel, head to head, randomized controlled diagnostic accuracy trial of cardiovascular magnetic resonance and myocardial perfusion scintigraphy. Eur Heart J Cardiovasc Imaging 2018;19:369–77. 29447342 10.1093/ehjci/jex342

[47] Lu M, Wang S, Sirajuddin A, Arai AE, Zhao S. Dynamic stress computed tomography myocardial perfusion for detecting myocardial ischemia: a systematic review and meta-analysis. Int J Cardiol 2018;258:325–31. 29433968 10.1016/j.ijcard.2018.01.095

[48] So A, Wisenberg G, Islam A, Amann J, Romano W, Brown J et al Non-invasive assessment of functionally relevant coronary artery stenoses with quantitative CT perfusion: preliminary clinical experiences. Eur Radiol 2012;22:39–50. 21938441 10.1007/s00330-011-2260-x

[49] Kido T, Kurata A, Higashino H, Inoue Y, Kanza RE, Okayama H et al Quantification of regional myocardial blood flow using first-pass multidetector-row computed tomography and adenosine triphosphate in coronary artery disease. Circ J 2008;72:1086–91. 18577816 10.1253/circj.72.1086

[50] Meinel FG, Pugliese F, Schoepf UJ, Ebersberger U, Wichmann JL, Lo GG et al Prognostic value of stress dynamic myocardial perfusion CT in a multicenter population with known or suspected coronary artery disease. AJR Am J Roentgenol 2017;208:761–9. 28177653 10.2214/AJR.16.16186

[51] van Assen M, De Cecco CN, Eid M, von Knebel Doeberitz P, Scarabello M, Lavra F et al Prognostic value of CT myocardial perfusion imaging and CT-derived fractional flow reserve for major adverse cardiac events in patients with coronary artery disease. J Cardiovasc Comput Tomogr 2019;13:26–33. 30796003 10.1016/j.jcct.2019.02.005

[52] Nous FMA, Geisler T, Kruk MBP, Alkadhi H, Kitagawa K, Vliegenthart R et al Dynamic myocardial perfusion CT for the detection of hemodynamically significant coronary artery disease. JACC Cardiovasc Imaging 2022;15:75–87. 34538630 10.1016/j.jcmg.2021.07.021 PMC8741746

[53] Flohr T, Schmidt B, Ulzheimer S, Alkadhi H. Cardiac imaging with photon counting CT. Br J Radiol 2023;96:20230407. 37750856 10.1259/bjr.20230407 PMC10646663

[54] Stalhammar F, Aurumskjold ML, Meyer S, Wiklund M, Wingren P, Liuba P et al Photon-counting computed tomography for paediatric congenital heart defects yields images of high diagnostic quality with low radiation doses at both 70 kV and 90 kV. Pediatr Radiol 2024;54:1187–96. 38700554 10.1007/s00247-024-05939-z PMC11182870

[55] Pontone G, De Cecco C, Baggiano A, Guaricci AI, Guglielmo M, Leiner T et al Design of CTP-PRO study (impact of stress cardiac computed tomography myocardial perfusion on downstream resources and PROgnosis in patients with suspected or known coronary artery disease: a multicenter international study). Int J Cardiol 2019;292:253–7. 31230938 10.1016/j.ijcard.2019.06.012

